# Recommendation to Reduce Patients’ Blood Pressure and Cholesterol Medication Costs

**DOI:** 10.5888/pcd12.150253

**Published:** 2015-11-25

**Authors:** Jonathan E. Fielding, Barbara K. Rimer, Robert L. Johnson, C. Tracy Orleans, Ned Calonge, John M. Clymer, Karen Glanz, Ron Z. Goetzel, Lawrence W. Green, Gilbert Ramirez, Nicolaas P. Pronk, the Community Preventive Services Task Force

**Affiliations:** Author Affiliations: Jonathan E. Fielding, University of California, Los Angeles, California; Barbara K. Rimer, University of North Carolina, Chapel Hill, North Carolina; Robert L. Johnson, Rutgers, The State University of New Jersey, Newark, New Jersey; C. Tracy Orleans, Robert Wood Johnson Foundation, Princeton, New Jersey; Ned Calonge, The Colorado Trust, Denver, Colorado; John M. Clymer, National Forum for Heart Disease and Stroke Prevention, Washington, DC; Karen Glanz, University of Pennsylvania, Philadelphia, Pennsylvania; Ron Z. Goetzel, Johns Hopkins University, Baltimore, Maryland; Lawrence W. Green, University of California, San Francisco, California; Gilbert Ramirez, Texas A&M University, College Station, Texas; Nicolaas P. Pronk, HealthPartners Institute for Education and Research, Minneapolis, Minnesota.

## Task Force Finding

The Community Preventive Services Task Force recommends reducing patient out-of-pocket costs (ROPC) for medications to control high blood pressure and high cholesterol when combined with additional interventions aimed at improving patient–provider interaction and patient knowledge, such as team-based care with medication counseling, and patient education.

This recommendation is based on strong evidence of effectiveness in improving medication adherence and outcomes for high blood pressure and cholesterol. Limited evidence was available to assess the effectiveness of reducing patient out-of-pocket costs for behavioral counseling or behavioral support services independent of reducing patient costs for medications. A summary of the Task Force finding and rationale is at www.thecommunityguide.org/cvd/ROPC.html.

## Definition

Reducing out-of-pocket costs (ROPC) for patients with high blood pressure and high cholesterol involves program and policy changes that make medications for cardiovascular disease (CVD) prevention more affordable. Costs for treatment medications can be reduced by providing new or expanded treatment coverage and lowering or eliminating patient out-of-pocket expenses (eg, copayments, coinsurances, deductibles).

Reducing out-of-pocket costs is coordinated through the health care system, and preventive services may be delivered in clinical or nonclinical settings (eg, worksite, community). ROPC can be implemented alone or in combination with additional interventions to enhance patient–provider interaction such as team-based care, medication counseling, and patient education. Program and policy changes may be communicated to patients and providers using targeted messages to increase awareness and use of covered services.

## Basis of Finding

The Task Force finding is based on evidence from 18 studies (published from January 1980 to July 2015) that assessed the effectiveness of reducing out-of-pocket costs for medications to treat high blood pressure, high cholesterol, or both (1). All 18 studies evaluated programs or policies that reduced patient out-of-pocket costs for medications to treat high blood pressure or high cholesterol.

Ten studies combined ROPC for medications with one or more additional interventions including team-based care with medication counseling (7 studies), proactive follow-up (5 studies), linkages to other resources and services (4 studies), disease management (3 studies), and patient education (4 studies). Nine of 18 studies assessed the impact of ROPC for medications on blood pressure and cholesterol outcomes. Six studies assessed the impact of ROPC on adherence to blood pressure- and cholesterol-lowering medications. Only one of 18 studies evaluated the impact of both medication adherence and blood pressure and cholesterol outcomes. Twelve studies were policy-based; 7 of these evaluated value-based insurance design (VBID).

The Task Force finding reflects 1) the focus of available studies on ROPC for medications, 2) meaningful improvements in blood pressure and cholesterol outcomes (median decrease of 5.9 and 3.75 mm Hg in systolic and diastolic blood pressure, respectively, in 4 studies and a reduction of 15 mg/dL in 1 study) in patients from studies in which most ROPC efforts were combined with additional interventions such as team-based care with medication counseling, 3) modest improvements in medication adherence (median adherence for all 15 blood pressure and lipid-lowering medications increased 3.0 percentage points in 6 studies) in studies with ROPC policy changes, and 4) the lack of studies including or evaluating ROPC for behavioral counseling or behavioral support services for patients with high blood pressure or high cholesterol, independent of ROPC for medications.

## Applicability

Fifteen of the 18 studies were conducted in the United States with study populations that included working-age adults balanced by sex. Studies examined outcomes in different racial and ethnic groups (ie, Hispanic, white, and African American) with similar results. Six studies found effectiveness of ROPC in improving treatment outcomes for low-income patients. Overall, results indicate that ROPC is effective in a wide range of patients with high blood pressure and high cholesterol in the US health care system. Evidence also shows applicability to diverse policy and program implementers, such as employers and government agencies.

ROPC can be coordinated with other interventions (eg, medication counseling) with the goal of increasing opportunities for patient–provider interaction on treatment issues such as medication side effects. No harms to patients from these interventions were identified in the included studies or the broader literature.

## Economic Evidence

The systematic economic review of the intervention included 9 studies that evaluated ROPC for medications to treat high blood pressure or high cholesterol. Seven of these were for reductions in medication costs as part of VBID plans. Two of the 9 studies combined reduced cost for medications with team-based care and 3 studies combined reduced medication cost with coaching for lifestyle or disease management. However, only one of the studies of these combined interventions provided the cost to implement both the ROPC and the added component.

Five of the 7 studies that estimated the effect of the intervention on nonpharmacy health care cost indicated these costs were reduced. The time frame of these analyses ranged from 5 years of follow-up to 1 year, with most in the 1- to 2-year range. Three studies that assessed net benefit of change in health care cost minus intervention cost indicated mixed results, one showing the intervention was cost-neutral and 2 indicating they were cost-increasing. No studies reported cost-effectiveness outcomes. An overall economic conclusion about the intervention cannot be drawn from this small and inconsistent body of cost-benefit evidence.

## Considerations for Implementation

This Task Force finding supports incorporation of policies or programs to reduce or eliminate out-of-pocket costs for medications to treat patients with high blood pressure or high cholesterol as one part of an effort to prevent cardiovascular disease. Team-based care and disease management programs were common additional interventions evaluated in the reviewed studies; broader health system efforts such as Patient-Centered Medical Homes could also provide a useful infrastructure to coordinate prevention activities. In addition, partnerships with employers, providers, and community-based organizations may provide resources and settings that enhance access to and use of preventive services.

Potential implementers include health care providers and plans, government agencies, and self-insured and fully insured employers. Review results suggest opportunities for innovative application of ROPC policies, coordination of programs, and partnerships for delivery of services. Linking medical and pharmacy claims data and other information systems across settings may enhance coordinated service delivery, monitoring of service use, and assessment of program effectiveness for multiple outcomes of interest.

To increase awareness and use of ROPC covered services, it is critical to promote ROPC benefits to patients and providers. Only 3 of 18 included studies described communicating available benefits for reducing out-of-pocket medication costs to patients via letter, newsletter, or company intranet. No reviewed studies evaluated or reported changes in awareness resulting from activities to communicate ROPC benefits.

Low-income patients experienced improved blood pressure and cholesterol outcomes after being treated with a combination of interventions including ROPC for medications. Implementers should consider promotion strategies that are innovative, culturally appropriate, and targeted to increase awareness among low-income groups with low medication adherence. Partnering with community organizations can also provide opportunities to increase awareness and use of ROPC benefits among underserved populations.

One ROPC policy approach is to reduce or eliminate copayments for generic medications. Providers may need to discuss appropriate generic medications with their patients. Prescribing providers can be important advocates for patients unaware of ROPC benefits. Providers can 1) ask patients about their ability to pay for medications and 2) be familiar with medications covered by patients’ health insurance plans and the costs to patients.

Reducing out-of-pocket costs for patients with high blood pressure and high cholesterol can be implemented as part of a broader effort to increase use of effective cardiovascular disease preventive services. Evidence in the review, including studies evaluating VBID, indicates ROPC interventions are effective in increasing adherence to medications in patients with different cardiovascular risk conditions. A comprehensive approach, for example, could coordinate ROPC for medications to improve blood pressure and cholesterol outcomes with evidence-based tobacco cessation treatments, coverage to improve management of patients with diabetes, or both. The Affordable Care Act, recognizing the potential of VBID plans to improve patient receipt of preventive health services without cost-sharing (2), provides opportunities to reduce patients’ out-of-pocket costs and assist in preventing cardiovascular disease through a section of the law featuring VBID and calling for health plan coverage of preventive health services (3).

## Evidence Gaps

Although evidence indicates effectiveness of ROPC for medications to control high blood pressure and high cholesterol, additional research should take into account the assessment of ROPC in other areas of cardiovascular disease preventive services (eg, behavioral counseling), especially when coordinated with ROPC for medications. Future studies should also describe efforts to effectively communicate the presence and availability of covered ROPC benefits and evaluate both the reach and effectiveness of different communication techniques. Relationships between cost reduction and patient use must be examined, providing evidence on thresholds and differential effectiveness. In addition, research could examine effectiveness of ROPC by total medication cost, proportional cost-reduction, patient income, or drug patent type.

In general, policy studies included in this review examined the impact of adding ROPC for medications for an entire patient population but evaluated only changes in medication adherence. Conversely, the studies evaluating multicomponent programs that include ROPC for medications examined clinical outcomes for patients in the program but did not report on changes in medication adherence. Both outcomes provide useful information to potential implementers and should be reported together.

There are very few complete economic evaluations of ROPC interventions for cardiovascular disease prevention services. Less than half of the interventions evaluated for effectiveness included any assessment of economic costs or benefits. Cost-effectiveness could not be calculated for VBID plans because their evaluations did not report clinical outcomes such as changes in blood pressure. The cost of communicating the ROPC benefits to providers and patients was not discussed or estimated in any of the economic studies.

Research efforts in these areas can improve understanding of the ways in which ROPC for medications to treat high blood pressure, high cholesterol, or both can help improve patient health.
